# The Evolution of Social Orienting: Evidence from Chicks (*Gallus gallus*) and Human Newborns

**DOI:** 10.1371/journal.pone.0018802

**Published:** 2011-04-20

**Authors:** Orsola Rosa Salva, Teresa Farroni, Lucia Regolin, Giorgio Vallortigara, Mark Henry Johnson

**Affiliations:** 1 Center for Mind/Brain Sciences, University of Trento, Rovereto, Italy; 2 Department of Developmental Psychology, University of Padova, Padova, Italy; 3 Centre for Brain and Cognitive Development, Birkbeck, University of London, London, United Kingdom; 4 Department of General Psychology, University of Padova, Padova, Italy; Cajal Institute, Consejo Superior de Investigaciones Científicas, Spain

## Abstract

**Background:**

Converging evidence from different species indicates that some newborn vertebrates, including humans, have visual predispositions to attend to the head region of animate creatures. It has been claimed that newborn preferences for faces are domain-relevant and similar in different species. One of the most common criticisms of the work supporting domain-relevant face biases in human newborns is that in most studies they already have several hours of visual experience when tested. This issue can be addressed by testing newly hatched face-naïve chicks (*Gallus gallus*) whose preferences can be assessed prior to any other visual experience with faces.

**Methods:**

In the present study, for the first time, we test the prediction that both newly hatched chicks and human newborns will demonstrate similar preferences for face stimuli over spatial frequency matched structured noise. Chicks and babies were tested using identical stimuli for the two species. Chicks underwent a spontaneous preference task, in which they have to approach one of two stimuli simultaneously presented at the ends of a runway. Human newborns participated in a preferential looking task.

**Results and Significance:**

We observed a significant preference for orienting toward the face stimulus in both species. Further, human newborns spent more time looking at the face stimulus, and chicks preferentially approached and stood near the face-stimulus. These results confirm the view that widely diverging vertebrates possess similar domain-relevant biases toward faces shortly after hatching or birth and provide a behavioural basis for a comparison with neuroimaging studies using similar stimuli.

## Introduction

Evidence from several different species has led to the proposal that some newborn vertebrates, including humans, have visual predispositions to attend to the head regions and motion pattern of conspecifics or of animate creatures in general [1]–[8]. In fact, such preferential attention to socially relevant stimuli often extends to other species. For example, chicks' early social preferences are clearly characterized by the absence of species specificity. This means that chicks' choices are not selective for their own species, but rather seem to be devoted to the individuation of any animate creature [1], [4]. Thus, chicks do not show any preference between a point light display representing the motion pattern of a walking cat and a point light display representing the motion pattern of a walking hen. Both stimuli, on the contrary, are preferred to non-biological motion displays [4]. Moreover, many studies demonstrated that naïve chicks preferentially approach conspecifics' (stuffed) heads (see Table 1). However, this preference extends to stuffed specimens of other species (a gadwall duck and polecat) [1]. A similar result has been obtained for monkeys: in the absence of prior experience Japanese macaques spend equal time looking at human or monkey faces. Again, both kind of faces are preferred over inanimate objects [7].

**Table 1:** Newly hatched domestic chicks.

	Experiment number	Stimuli	Properties controlled for	Main result
Rosa Salva et al. (2010) Dev Sci, 13, 565–577.	Exp. 1, 3–4	Schematic faces	Symmetry along the vertical axis, up-down distribution of inner elements, object-like structure	Preference for approaching face-like stimuli, independently of the number of features present in the upper vs. lower half of the configuration
Bolhuis & Horn (1997) Physiol Behav, 62, 1235–2139.	Exp. 1, 2	Naturalistic object (stuffed jungle fowl) vs. artificial object (red box)	Presence of structured objects, approximate vertical symmetry	Preference for approaching the naturalistic object.The emergence of the preference is characterized by a sensitive period that can be delayed by injections of anaesthetic agents (equithesin)
Hampton et al. (1995) Behaviour, 132, 451–477.	Exp. 1–3	Naturalistic object (stuffed jungle fowl) vs. artificial object (red box)	Presence of structured objects, approximate vertical symmetry	Preference for approaching the naturalistic object, emerging 2–5 h after non-specific releasing experience (motor activity, handling, exposure to maternal calls etc)
Davies et al. (1992) Dev Psychobiol, 25, 251–259.	/	Naturalistic object (stuffed jungle fowl) vs. artificial object (red box)	Presence of structured objects, approximate vertical symmetry	Preference for approaching the naturalistic object (characterized by a sensitive period that can be delayed by administration of neurotoxin DSP4)
Johnson & Horn (1988) Anim Behav, 36, 675–683.	Exp. 1–5	Naturalistic objects (stuffed jungle fowl, gadwall duck and polecat); altered versions of the stuffed fowl (disarticulate fowls maintaining or removing outline complexity, scrambled fowls preserving only the texture of the original stimuli) and artificial objects (simple red box and striped red box)	Presence of structured objects, approximate vertical symmetry, stimulus complexity, stimulus outline, stimulus texture	Preference for approaching the normal stuffed jungle fowl with respect to both the simple and the complex artificial stimulus, and to a scrambled fowl that maintains only the texture of the naturalistic stimulus.Disarticulated fowls reassembled in anatomically unusual ways (either preserving outline complexity or mounting the limbs on a square cardboard background) and other stuffed animals are equally preferred to the normal stuffed hen. The head alone of the stuffed fowl elicits a similar preference to the whole hen, indicating that features in the head region are crucial for chicks' approach behaviour
Bolhuis & Trooster (1988) Anim Behav, 36, 668–674.	/	Naturalistic object (stuffed jungle fowl) vs. artificial object (a red box whose overall attractiveness is manipulated changing its illumination level)	Presence of structured objects, approximate vertical symmetry	After imprinting on an artificial stimulus, subsequent exposure to a naturalistic object determines a shift in chicks' preference in favour of the latter (such secondary imprinting is not evident in chicks first exposed to the stuffed hen and then to the red box)
Bolhuis et al. (1985) Dev Psychobiol, 18, 299–308.	Exp. 1, 2	Naturalistic object (stuffed jungle fowl) vs. artificial object (red box)	Presence of structured objects, approximate vertical symmetry	Preference for approaching the naturalistic object; the emergence of the preference is speed up by exposure to visual patterned input (abstract geometrical configuration)
Johnson et al. (1985) Anim Behav, 33, 1000–1006.	/	Naturalistic object (stuffed jungle fowl) vs. artificial object (red box)	Presence of structured objects, approximate overall vertical symmetry	24 h after imprinting on either the naturalistic or the artificial object, chicks prefer to approach the naturalistic object, regardless of their imprinting stimulus (the preference emerges also after simple motor activity)
Boakes & Panter (1985) Anim Behav, 33, 353–365.	Exp. 2	Live hen, artificial moving objects (rotating cup, windmill)	Presence of structured objects, approximate vertical symmetry	After imprinting on a live hen, no secondary imprinting on an artificial object is possible

While different species depend on different sensory modalities, faces have had great adaptive relevance for many social species throughout evolution from ancestral past to the present. Attention to faces allows individuals of a species to identify living things, such as conspecifics, to recognize different individuals, to engage in social interaction with them, and in some cases to obtain information about their intentions, emotions and attentional or motivational state. As will be further discussed below, evidence supports the existence of specific biases for the visual processing of faces compared to other objects, probably due to their great adaptive relevance for social animals (e.g. [9]–[13]). This implies that similar mechanisms may be present in different social species allowing for preferential attention toward, and processing of faces shortly after birth [14].

With regard to faces, the more specific claim is that these newborn preferences are (i) domain-relevant to the extent that other naturally occurring stimuli do not draw attention in the same way, (ii) are not based on rapid early learning, but are present from birth/hatching, and (iii) may be common to many vertebrates (this is supported also by the fact that face preferences are not species-specific). In the present study we directly assess these claims by testing both newly hatched chicks (*Gallus gallus*) and human newborns with the same face and visual noise stimuli matched for some of their psychophysical properties (such as spatial frequencies and colour distribution). On the one hand, the use of *identical* stimuli for both species is necessary to perform a direct comparison between human newborns' and domestic chicks' data (a primary aim of the present study). On the other hand, this implies the use of human faces as stimuli for domestic chicks. Human faces vs. frequency matched images have already been employed in neuroimaging studies on human infants [15], [16]. However, newborns' responses to such stimuli have never been tested behaviourally. Thus, a crucial point for the present study is to bridge the gap between behavioural and neuroimaging studies. Moreover, we also want to create a direct parallel between human newborns' and domestic chicks' data.

It should also be considered that the use of human faces as stimuli for chicks is broadly justified by the existing literature. In fact, previous studies demonstrated the non-species specific nature of newborns' social preferences (see above). This empirical finding is also theoretically consistent with the assumptions of one of the leading theories about face preferences in newborn vertebrates. In fact, there is general agreement that the CONSPEC-CONLERN model is one of the more successful theories in accounting for the early experience-independent social preferences of newborn vertebrates. According to this model, during the first days of life, a subcortical template-matching device, called CONSPEC, is responsible for orienting newborns' attention toward faces. In particular, CONSPEC responds to stimuli that match an unlearned representation of faces' structure (see [3]). This mechanism is present in different species and it is highly adaptive, since it ensures early preferential attention to animate creatures. It should be noted that, in the environment of the newborn individual, such animate creatures will mainly consist of conspecifics and caretakers. Thus, CONSPEC ensures the formation of appropriate social bonds with conspecifics (e.g. imprinting on the mother hen or siblings, for domestic chicks). Moreover, at least for human beings, CONSPEC provides extensive exposure to faces during sensitive periods of cortical development (contributing to the specialization of cortical areas for face processing, see [17]). In this framework, early face preferences are by definition supposed to be not species specific: the CONSPEC mechanism codifies only an extremely broad representation of faces' structure (mainly consisting of 3 dark blobs in a triangular arrangement on an oval-shaped background, see [17]). Such a representation, sufficient to detect faces and discriminate them from most stimuli encountered in the natural environment, can not support selectivity for conspecifics' faces. This kind of selectivity will be acquired later through experience, thanks to a learning device called CONLERN. This mechanism supports the recognition of individuals by encoding their peculiar features (in chicks CONLERN is responsible for the recognition of the imprinting object with respect to other conspecifics) (see also [7] for evidence of experience-driven species selectivity in monkeys; similar findings in humans concern the ontogenetic development of the other species effect, [18], [19]).

Previous studies already assessed the role of a good number of potentially relevant perceptual properties of stimuli in determining face preferences of newborn babies [2], [3], [20], [21] or domestic chicks [1], [8] (for a partial summary of the available literature see Tables 1 and 2). In fact, the present paper builds on a rich and substantial literature demonstrating the robustness and domain specificity of face preferences in both the species studied. In these previous works, stimuli were controlled for vertical symmetry. Depending on the kind of stimuli employed, they presented either perfect symmetry (that can be obtained in artificial images), or approximate overall symmetry (characterising real objects and their photographic images). Other perceptual properties, whose role has already been clarified, are: presence of structure and of object-looking parts, up-down asymmetry in the distribution of inner elements, contrast polarity, direction of illumination, overall brightness and visibility, stimulus texture, stimulus complexity and stimulus outline (see Tables 1 and 2).

**Table 2:** Newborn human babies.

	Experiment number	Stimuli	Properties controlled for	Main result
Farroni et al. (2005) PNAS, 102, 17245–17250.	Exp. 1a, 1b	Schematic faces	Symmetry along the vertical axis (non-face stimuli are constructed rotating the inner face features by 180° with respect to the outer facial contour), contrast polarity	Preference for looking at face-like stimuli only in images having the normal contrast polarity expected for a face
	Exp. 2a, 2b	Photographic images of faces	Approximate symmetry along the vertical axis, contrast polarity, direction of illumination	Preference for looking at face-like stimuli only in images having the normal contrast polarity expected for a face or illuminated from above (natural illumination)
Macchi Cassia et al. (2004) Psychol Sci, 15, 379–383.	Exp. 1–3	Photographic images of faces	Approximate symmetry along the vertical axis (in Exp. 1 the non-face stimulus is obtained rotating the inner face features by 180°), presence of structured object-like visual patterns	Preference for looking at naturally arranged faces and at the visual pattern with more high contrast elements in its upper part
Farroni et al. (2004) Infancy, 5, 39–60.	Exp. 1	Schematic faces	Presence of structured object-like visual patterns	Preference for looking at faces with direct gaze, more resembling the “canonical” representation of face's structure hypothesised to guide newborns' face preferences (as opposed to adverted gaze)
Farroni et al. (2002) PNAS, 99, 9602–9605.	Exp. 1	Photographic images of faces	Presence of structured object-like visual patterns	Preference for looking at faces with direct gaze (see above)
Macchi Cassia et al. (2001) Dev Sci, 4, 101–108.	/	Schematic faces	Symmetry along the vertical axis (the non-face stimulus is obtained rotating the inner face features by 180°)	Preference for looking at the schematic face
Batki et al (2000) Inf Behav Dev, 23, 223–229.	/	Photographic images of faces	Approximate symmetry along the vertical axis, presence of structured object-like visual patterns	Preference for looking at faces with open eyes, more resembling the “canonical” representation of face's structure hypothesised to guide newborns' face preferences (as opposed to faces with eyes closed)
Farroni et al. (1999) Dev Sci, 2, 174–186.	Exp 1, 4	Schematic faces	Symmetry along the vertical axis (the non-face stimulus is obtained rotating the inner face features by 180°)	A face-like stimulus (but not a non-face-like one) is effective in engaging a subcortical collicular visual mechanism that determines the presence of a gap effect (facilitation in disengagement from a central fixation if a temporal gap is introduced between its disappearance and the appearance of a peripheral fixation point).
Simion et al. (1998) J Exp Psychol Human, 24, 1399–1405.	Exp. 1	Schematic faces	Symmetry along the vertical axis (the non-face stimulus is obtained rotating the inner face features by 180°)	Preference for looking at the schematic face (selective for stimuli presented in the temporal hemifield)
Slater et al. (1998) Inf Behav Dev, 21, 345–354.	Exp. 1, 2	Photographic images of faces (rated for their attractiveness by adults)	Approximate symmetry along the vertical axis, presence of structured object-like visual patterns, attractiveness. In Exp. 2 stimuli are also equated for brightness and contrast.	Preference for looking at attractive faces, more resembling the “canonical” representation of face's structure hypothesised to guide newborns' face preferences (as opposed to unattractive ones)
Valenza et al. (1996) J Exp Psychol Human, 22, 892–903.	Exp. 1a, 1b, 3	Schematic faces	Symmetry along the vertical axis (the non-face stimulus is obtained rotating the inner face features by 180°), visibility of the stimuli to newborns' visual system	Preference for looking at the schematic face (even when compared to stimuli having the optimal visibility for newborns' visual system)
Umiltà et al. (1996) Europ Psychol, 1, 200–205.	Exp. 1, 3, 4	Schematic faces	Symmetry along the vertical axis (the non-face stimulus is obtained rotating the inner face features by 180°), visibility of the stimuli for newborns' visual system	Preference for looking at the schematic face. The preference for the face is evident even when compared to stimuli having the optimal visibility for newborns' visual system, but is selective for stimuli presented in the temporal hemifield (index of subcortical engagement).
Johnson et al. (1991) Cognition, 40, 1–19.	Exp. 1–2	Schematic faces (represented with different levels of detail)	Symmetry along the vertical axis (non-face stimuli are obtained rotating the inner face features by 180° or displacing the features in unnatural positions, preserving overall symmetry)	Preference for looking at naturally arranged schematic faces
Goern et al. (1975) Pediatrics, 56, 544–549.	/	Schematic faces	Symmetry along the vertical axis (severely and moderately scrambled non-face stimuli are obtained by displacing schematic face features in unnatural positions, preserving overall symmetry); overall brightness	Preference for looking at naturally arranged schematic faces

In particular, in chicks, research using naturalistic stimuli and real objects demonstrated a preference for the configuration of features contained in the head of a hen using, among others, stimuli that were controlled for texture, outline, stimulus complexity, presence of structure, object-like appearance and approximate symmetry (see Table 1). For example, it has been demonstrated that domestic chicks show an unlearned bias to approach a stuffed hen (jungle fowl) with respect to artificial objects with different degrees of complexity. This same bias in favour of the stuffed hen is evident also with respect to a control stimulus created by cutting the trunk pelt of a hen into small pieces and pasting them in random order on a box. This control stimulus has a neat, clearly visible and grossly symmetrical outline: due to the fact that the pieces of the hen's pelt are pasted over a rectangular box, its overall shape is rectangular. We believe that this control stimulus can be defined as “object-looking”, using the words of an anonymous referee. Obviously, the control stimulus also presents the same overall visual texture of the “canonical” intact hen (since its surface was covered with hen's pelt).

Appropriate controls revealed that chicks' preference for the intact hen was not due to its outline complexity, nor to the presence or anatomical plausibility of any other body part, except for the head region. In fact, the same level of preference was shown by chicks for a whole stuffed hen and for the simple head of the hen mounted upon a rotating box [1].

In addition, recent research using schematic stimuli confirmed a preference for face-like configurations in naïve chicks, controlling properties such as vertical symmetry and structure. The use of artificially constructed schematic images allowed a very precise control of vertical symmetry. Also in this case, chicks preferred to approach face-like stimuli even if paired with other equally structured, symmetrical and object-looking configurations [8]. The fact that other properties can act a stronger role than symmetry in driving chicks' preferences, is not completely surprising. This is, in fact, consistent with results obtained in previous studies, in which chicks preferred the grossly symmetrical stuffed hen to perfectly symmetrical artificial objects. In the same study, chicks did not show any preference for the same stuffed hen over patently asymmetrical disarticulated hens, provided that the hen's face was still visible [1]. It should also be considered that previous research [22] demonstrated that, under some circumstances, naïve chicks show a spontaneous preference for stimuli characterized by asymmetry (finding a complex interaction of this initial preference with experiential factors, indicative that symmetry per se is not necessarily preferred by our animal model, see [23]).

Since a wide range of control stimuli for faces have already been examined, we decided to concentrate our efforts on the role of other potentially relevant perceptual properties, namely, spatial frequency composition and colour distribution. Control stimuli that match faces in their spatial frequency composition have already been commonly used in research on the neural bases of face perception [15], [16]. However, to the best of our knowledge, this control stimulus has not yet been used to investigate behavioural preferences in newborn babies and domestic chicks. In the present study we are thus going to fill this gap between behavioural and neuroimaging studies. As pointed out by an anonymous reviewer, in future experiments it could be worth to explore several other potentially relevant perceptual properties of the stimuli. However, considered the overall evidence available at this stage of our investigation (see Tables 1 and 2), we believe that a control for spatial frequency content was needed and timely, and that the association between domestic chicks' and human newborns' data important. This association is also interesting for the interpretation of recent studies on the neural correlates of infants' face preferences [15], [16].

The study of face perception has been a primary battleground for the empirical investigation of nature-nurture issues in human development (for the role of experience in determining the “special” status of faces see [24], [25], [26]). While one group of researchers has generated evidence that human newborns have domain-relevant preferences for attending to faces, and indeed, specific aspects of faces such as direct-gaze [27], and happy expressions [28], other researchers have suggested that these effects can be explained by domain-general biases or the comparative visibility of stimuli. Particularly influential with regard to the latter view was the Linear System Model (LSM) [29] that attributed face preferences in young infants to the relative visibility of stimuli to an underdeveloped visual system. While LSM successfully accounted for some visual preferences in babies, it nevertheless failed to account for the full range of experimental data [30], [31]. More recently however, a neural model based on the tuning selectivity of visual neurons for spatial frequencies successfully simulated some of the experimental data on face-preferences at birth [32]. The control stimuli used in the current experiments are matched to faces in their spatial frequency composition, meaning that any preference for faces observed cannot be attributed to this psychophysical dimension.

One of the most common criticisms of the work supporting domain-relevant face biases in human newborns is that the majority of the studies conducted (with some notable exceptions) are with newborns of more than a few hours old. Thus, it remains possible that very rapid early learning contributes to the specificity of some of the effects observed [33]. A second criticism often aired about this body of work is that the sub-cortical circuits that dominate the control of human newborn behaviour may lack the specificity of processing required to influence face preference behaviour. These criticisms of the data from human newborns can be addressed by testing newly hatched visually-deprived chicks whose preference for visual stimuli can be assessed prior to any other visual experience with faces, a type of experiment obviously not possible with human newborns for ethical reasons. Further, indirect evidence suggests that visual predispositions in the chick are mediated by retino-tectal (“sub-cortical”) routes. For example, while several different localised forebrain regions impair stages of visual learning and consolidation, none of the lesions to date have effected the predisposition to orient to conspecifics [34]. The existence of similar visual preferences in the chick supports the idea that the equivalent routes in the primate brain may share a common function, and avian-mammal brain homologies have been increasingly recognized [35]. While data from chicks has been brought to bear on the human newborn literature for some time [3], [36], to date these have been separate sets of studies with different stimuli and test measures (e.g. [1], [3]).

In the present work, we directly compare two species (domestic chickens and humans) that, while phylogenetically distant and adapted to different ecologic niches, share some important traits. Both are highly social species and the selective pressures they have been exposed to, even though clearly different, could have led them to process faces in a privileged fashion, and to spontaneously prefer faces from shortly after birth. In fact, face features or a configuration of face features can be used [37], [38], and tend to be used [39], [40] by domestic chickens in order to recognize different individuals and to guide social interactions, in line with what is observed for our own species.

It is important to note a fundamental difference between newborn babies and newly-hatched chicks: the former are the offspring of a highly altricial species, whereas the latter are the offspring of a highly precocial species. This of course impels caution when hypothesising the presence of similar selective pressures acting on both species. In fact, it is also possible to hypothesise that a similar trait (i.e. a spontaneous preference for face-like configurations) could bring different adaptive advantages to the two species. For example, domestic chicks, that are ready to move away from their nest in the first days of life (being thus at risk of losing contact with their mother hen), could need to direct their attention toward conspecifics in order to avoid imprinting on inanimate features of the environment. Imprinting on the appropriate social object is likely to be a fundamental adaptation for this species in order to maintain brood cohesion. On the other hand, newborn babies, that are completely dependent from parents' care for their survival, need to establish and maintain the infant-caretaker relationship. The creation of appropriate social bonds could thus be one of the main survival needs of the offspring of an altricial species. These have been described as *ontogenetic adaptations* [41]. Moreover, preferential attention for faces in human newborns could serve to ensure an adequate level of exposure to faces to the still developing cerebral cortex, allowing for the development of cortical specialization for face processing. The latter is sometime referred to as a *deferred adaptation*. We suggest that the phylogenetic distance between the two species we have tested, as well as the many differences in their ecological niche, adds relevance to the comparison between the two species in our study.

We hypothesise that, contrary to claims of the uniquely primate nature of the specialization for face processing observed in humans (e.g. [42]), similar basic mechanisms could operate in different species due to the selective pressures shaping the early functions of the brain.

**Figure 1:**
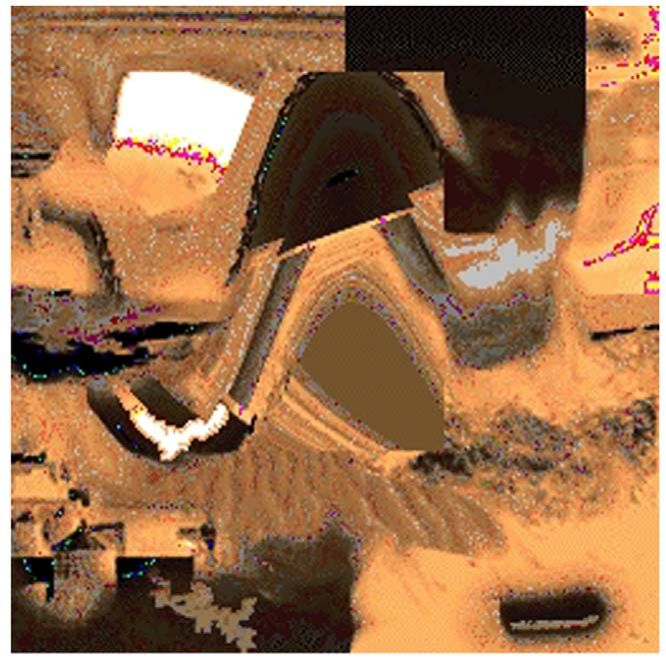
Example of one the control noise stimuli used in the newborns' study. The same stimulus reproduced in this figure was also used with chicks in Experiment 2. See also [15], [16] for the face stimuli employed.

In the present study, for the first time, we test the prediction that both newly hatched chicks and human newborns will demonstrate similar preferences for face stimuli over spatial frequency matched structured noise. Our first experiment investigated newborns' spontaneous preference for looking at a human face with respect to a simultaneously presented noise stimulus that was matched to the face in terms of the component spatial frequencies and colour distribution (Fig. 1) (see also [15], [16]). Similarly, in our second experiment we tested 2-day-old chicks' preferences for orienting their head and approaching the same human face and visual noise stimuli, that were simultaneously presented at the two opposite ends of a longitudinal runway [43]. The prediction was confirmed providing strong converging evidence that many vertebrates have a domain-relevant bias toward faces shortly after hatching or birth.

## Materials and Methods

### Experiment 1

#### Ethic Statement

The experimental procedures used to test human newborn participants were approved by the Ethical Committee of the University of Padova. Oral informed consent was obtained from the infants' parents before testing. Oral consent was chosen as it is a standard procedure for testing newborn babies, it reduces any possible inconvenience for the parents during newborn participants' selection and it allows to take advantage of the limited attention span and awake time of the newborn participants. The parents were always present at test and could interrupt it at any moment. This procedure was approved by the Ethical Committee.

#### Participants

Thirteen healthy, full-term newborns were selected from, and tested at, the maternity ward of the Pediatric Hospital of Monfalcone in Italy. All newborns were healthy and free of any known neurological or ocular abnormality, had been delivered normally, and received an Apgar score of at least 8 at 5 minutes. The age range at time of testing was between 24 and 120 hours. The testing took place only if the baby was awake and in an alert state.

#### Apparatus and Stimuli

The infant sat on the experimenter's lap, facing the midline of a grey screen. The experimenter holding the infant (a student) was blind with respect to the hypotheses under test. The eyes of the infant were aligned with a red flickering LED that was located in the centre of the screen and was used to attract the infant's gaze at the start of the trial. The stimuli were 5 pairs of images taken from [15] and [16]. Each pair of stimuli consisted in a full color image of a female human face (face stimulus) and a scrambled version of the same image (noise stimulus, Fig. 1) artificially constructed with the same spatial frequencies and colour as the corresponding face (see [16] for details). At a viewing distance of about 30 cm, each stimulus subtended about 23° of visual angle horizontally and vertically. The stimuli were projected on the screen at a distance of approximately 15° from the centre.

#### Procedure

Once the newborn was seated in front of the screen, as soon as she/he fixated the centre of the screen, the experimenter (who watched the newborn's eyes via a video monitor system) initiated a trial and presented the stimuli on the screen. The stimuli remained on for as long as the infant fixated one of them (infant control procedure). When the infant shifted her/his gaze away from the display for more than 10 sec, the experimenter removed the stimuli and presented the next trial. In the second trial the location of the stimuli was reversed. Videotapes of the baby's eye movements throughout the trial were subsequently analyzed by two coders blind as to the location of noise and human face stimuli. The coders recorded, separately for each stimulus and each trial, the number of orienting responses and the total fixation time. The inter-rater reliability for 10% of the total participants was high (Cohen's Kappa  = 0.87 for the duration of fixation and 0.95 for the number of orientations). While the coders could see the corneal reflection of the stimuli, they were blind to the hypothesis tested.

#### Data analysis

Dependent variables considered were (i) the number of orienting responses (i.e. fixations) directed at the two stimuli, (ii) the total amount of time spent fixating each one of the two stimuli for the whole length of the test. To represent the percentage of gaze orienting responses performed toward the face stimulus, an index was calculated from the number of orienting responses performed toward the two stimuli, using the formula:

(Orienting responses toward human face/Orienting responses toward human face + Orienting responses toward noise) X 100.

To represent the proportion of looking time spent fixating the human face stimulus, a similar index was calculated from the time spent looking at the two stimuli using an analogous formula.

Significant departures from chance level (50%), which indicated a preference for the human face stimulus (>50%) or noise stimulus (<50%), were estimated by one-sample two-tailed t-test for both indexes.

### Experiment 2

#### Ethics Statement

This experiment complies with the current Italian and European Community laws for the ethical treatment of animals and the experimental procedures were licensed by the Ministero della Salute, Dipartimento Alimenti, Nutrizione e Sanità Pubblica Veterinaria (permit number 08939/SSA). Due to the observational and not invasive procedures employed, the present experiment involved only minimal discomfort for the animals.

#### Subjects

Subjects were 40 (20 males and 20 females) domestic chicks (Gallus gallus domesticus) of the “Hybro” strain (derived from the White Leghorn) hatched, reared and tested within the Comparative Psychology Laboratory, Department of General Psychology, University of Padova. Fertilized eggs were obtained weekly from a local commercial hatchery (Agricola Berica, Montegalda (VI), Italy) on the 14^th^ day of incubation. Eggs were incubated (MG 70/100 Rurale incubator) from Days 14 to 17. On the 17^th^ day of incubation eggs were placed in a hatchery (MG 100). During incubation and hatching (which took place on the 21^st^ day of incubation) eggs and chicks were maintained in complete darkness.

#### Rearing conditions

After hatching in the darkness chicks were immediately placed singly in metal home-cages (28 cm×16 cm×40 cm), lit (24 h/day) by 36 W fluorescent lamps placed 15 cm above the cages. Cages' walls and floor were lined with white opaque paper. Chicks were maintained at a controlled temperature (c. 28–31°C) and humidity (c. 70%), with water available ad libitum. No reflection from the water surface was visible. At the beginning of the first day of life, some food was scattered over the floor in each cage. Care was taken in order to avoid chicks receiving any visual experience concerning faces, prior to the moment of the test. In particular, chicks never saw the experimenter's face or the face of another chick. Whenever required chicks were transported in complete darkness. Chicks were kept inside a closed cardboard box when transported. Manipulation of the chicks for sexing and daily care was performed only after covering the chick's head or eye region, preventing it from any possible visual experience.

#### Apparatus

The test apparatus consisted of a white-plywood longitudinal runway, from here on named the ‘choice-runway’, with the two experimental stimuli being presented at the two opposite ends. The choice runway was divided into three sectors: a central area that was equidistant from the two experimental stimuli, and two side-areas, each of them adjacent to one of the two stimuli. The dimensions of the apparatus were as follows: choice-runway 45 cm long×22.3 cm large, 30 cm high; central sector 15 cm long; two lateral sectors 15 cm long each.

Each side-area ended with a translucent glass screen. The two stimuli were placed upon these glass partitions. Each stimulus and the inner area of the choice runway was lit by a 40 W lamp placed beyond the glass partition, while the rest of the experimental room was maintained in darkness. The two stimuli were placed upon the glass partitions so that their lower boundary was at 1 cm of height from the level of the runway floor. A video camera was placed above the apparatus, so that we could record the chick's behaviour during the test. The camera was also connected to a monitor screen in the same room, enabling the experimenter to score behaviour on-line during test, without disturbing the animal.

#### Test stimuli

The two test stimuli employed the present experiment represented a full colour image of a human face and a noise stimulus (scrambled face, see Fig. 1) constructed with the same spatial frequencies and colour distribution as the face. For a discussion of issues concerning the use of human faces as stimuli in this experiment, see the Introduction (see the Introduction also for a description of the wide range of previous studies justifying the choice of frequency-matched noise stimuli). It should be noted that, for chicks, the small irregular shapes with imperfect symmetry composing the noise stimulus could, in principle, result more attractive than the face-stimulus (hypothesising the absence of any preference for face-like configurations). For example, some of the features composing the noise stimulus could present the appropriate size and configuration to elicit feeding responses (the animals, if not otherwise motivated by the presence of the face stimulus, would probably have searched for food in the test apparatus).

The two stimuli were one of the five pairs of stimuli employed in the human newborn experiment, which had been previously used in two human neuroimaging studies [15], [16]. The decision to use a single pair of stimuli in this experiment was motivated by two considerations. Firstly, human babies data (Experiment 1) did not reveal any evidence of differential responses to the 5 pairs of stimuli (even though sample size did not allow a formal analysis on this regard). Second, due to the highly schematic and generic representation of social object encoded by CONSPEC, any stimulus presenting the correct (face-like) configuration of features should elicit comparable preferences, irrespective of its individual and idiosyncratic characteristics. As discussed above, the non-specific nature of the representation guiding chicks' preferences had already been proven in previous studies, using both schematic stimuli [8] and naturalistic objects [1] (see the Introduction and Table 1). Thus, in order to minimize the number of animals to be used in experiments, it seemed reasonable to limit the number of stimuli.

From the chick's starting point at the centre of the apparatus each stimulus subtended about 22° of visual angle horizontally and vertically.

#### Procedure

The test was performed on the second day of life. Each subject was carried, in a closed cardboard box, to the experimental room (located near the rearing room, and kept at 29–30°C with a humidity of 68%), where the chick was placed directly in the central area of the test apparatus. The chick's position at the starting point with respect to the test stimuli, as well as the position of the two stimuli within the apparatus, was balanced across animals.

Chicks' behaviour was recorded for a total of 6 consecutive minutes. If the chick remained in the mid compartment this indicated no choice, whereas entrance and presence of the chick in one of the side compartments was regarded as a preference for the object placed at that end of the runway (see [43] for initial validation of these procedures). A computer-driven event recorder allowed the experimenter to score the time (seconds) spent minute by minute by the chick in each of the three areas during the overall test period.

Moreover, to allow a more direct comparison with newborn data, orienting responses were also recorded. An orienting response was defined as a discrete head turning movement, which led the chick to fixate one of the two stimuli within its binocular central visual field. Operationally, this meant that an orienting response was scored whenever the chick directed the tip of its bill toward one of the two stimuli. In order to record an orienting response the following criteria were used: (i) the chick had to be motionless when the orienting response was performed (this mainly led to the exclusion of responses performed while the chick was walking); (ii) the starting-orientation of the head, before the beginning of the response, had to be equidistant from the two stimuli (i.e. none of the two stimuli had to be already fixated within the frontal binocular visual field before the beginning of the response); (iii) pecking responses which also induced a change in head orientation were not considered orienting responses.

#### Data analysis

Behavioural measures considered were: (i) first stimulus approached by each chick (i.e. the first side sector entered during test); (ii) percentage of orienting responses performed toward each stimulus on the overall number of orienting responses; (iii) percentage of time spent near the human face stimulus (i.e. of time spent in the lateral sector adjacent to the human face). All measures were scored with a blind procedure (i.e. the scorer was unaware of the aims of the research conducted). To compare the number of chicks that approached first the human face or the noise stimulus we used the chi-square test of independence. To represent the percentage of orienting responses performed toward the human face stimulus, an index was calculated from the number of orienting responses performed toward the two stimuli, using the formula:

(Orienting responses toward human face /Orienting responses toward human face + Orienting responses toward noise) X 100.

To represent the proportion of time spent near the human face stimulus, a similar index was calculated from the time spent into the two lateral sectors using a similar formula. Significant departures from chance level (50%), which indicated a preference for the human face stimulus (>50%) or noise stimulus (<50%), were estimated by one-sample two-tailed t-test for both indexes.

## Results

### Experiment 1

The percentage of gaze orienting responses performed by newborns toward the human face stimulus was significantly different from chance level (t_(12)_ = 3.663, p = 0.003, Mean  = 60.84%, SEM  = 2.96%). Also the percentage of time spent fixating the human face stimulus was significantly different from chance level (t_(12)_ = 5.576, p<0.001, Mean  = 78.40%, SEM  = 5.09%). Infants oriented their gaze more frequently toward the human face stimulus and spent more time looking at this same stimulus. Furthermore, a regression analysis on the percentage of the fixation time towards the faces against infant age did not show any developmental effect (R^2^ = 0; F_(1,11)_ = 0.005; p = 0.945) allowing us to infer that the effect is not being driven solely by the older newborns.

### Experiment 2

A significantly greater number of chicks approached the human face stimulus first (X^2^_1_ = 8.100, p = 0.004; 29 chicks approached the human face and 11 approached the noise stimulus). Moreover, both the percentage of orienting responses toward the human face stimulus and the percentage of time spent near the human face stimulus were significantly different from chance level (t_(39)_ = 2.999, p = 0.005, Mean  = 58.59%, SEM  = 2.86%; and t_(39)_ = 3.821, p = 0.000, Mean  = 71.13%, SEM  = 5.53% respectively). Chicks oriented more frequently toward and stayed longer near the human face stimulus.

## Discussion

In the present study we directly compared data from newly hatched chicks and human newborns to contribute to support the claim, together with previous studies (see Tables 1 and 2), that some vertebrate species have predispositions to attend to stimuli that resemble the faces of conspecifics, regardless of their spatial frequency composition. It has been claimed that faces have a “special” status in visual processing, due to their relevance in social life throughout evolution. From a developmental perspective, this means that newborns should be equipped with domain-relevant preferences (likely to engage attention on faces occurring in the natural environment), which are not learned and may be present in a similar form in different vertebrates. Such preferences do not need to be selective for individual identity, for breed or for species: this kind of discrimination will be learned thanks to post-natal visual experience naturally provided by the surrounding social environment (see the Introduction for a description of the role of CONLERN, a putative mechanism for that function). We tested this hypothesis by investigating preferences displayed by both newly hatched chicks and human newborns for human faces with identical stimuli and similar test measures. Specifically, we assessed the degree of domain-relevance of face preferences in the two species by comparing human face images to psychophysically-matched visual noise. Both species significantly preferred to orient toward human faces, approach human faces (chicks) or to observe human faces (chicks and human newborns) compared to the control stimuli. Numerous previous studies demonstrated similar and very robust preferences while controlling a good number of other visual properties of stimuli such as symmetry or presence of structure (e.g. [2], [8], [20]) (see Tables 1 and 2 and the Introduction for further details). However, this is the first comparative study that controls for the spatial frequency composition of the stimuli.

The results of the present paper reduce the likelihood that face-preferences in human newborns are based on very rapid learning during the first hours. As mentioned earlier, for practical and ethical reasons it is hard to rule out very rapid visual learning as a factor in human newborn studies. However, chicks tested in the present study showed exactly the same preference as newborns, but in the absolute absence of any prior visual experience with faces. Similar findings have been obtained in two previous studies with animals. The first study demonstrated a preference for schematic face-stimuli in visually deprived chicks [8]. However, while the control stimuli employed controlled for other low-level perceptual properties such as presence of structure and vertical symmetry, they were not matched for the spatial frequency. The second study demonstrated that visually deprived monkeys prefer faces to other objects [7]. However, methodological issues associated with that study could have affected the effectiveness of the deprivation procedure (e.g. frequent tactile exploration of their own faces performed by monkeys) and the control stimuli employed differed from faces in many different respects [7]. Moreover, no direct comparison with human data was presented in these previous studies. Thus, to the best of our knowledge, the present study is the first that directly compares preferences displayed by naïve animals and human newborns.

Another important advantage of the present study is that it bridges the gap between behavioural investigations of early social preferences and neuroimaging studies that assessed the neural correlates of human infants' face perception using a similar approach (i.e. comparing the activation observed for faces to the activation observed for frequency matched noise-stimuli). On the basis of the present work that reports behavioural responses to this kind of stimuli, future studies could also involve comparative investigations of the neural correlates of face preferences in domestic chicks and human newborns.

In this regard, it is also interesting to note that homologies in the brain structures of mammals and birds are being increasingly recognized [e.g. 35]. Specifically, three areas have been hypothesised to be part of the human subcortical face-detection route (Superior colliculus, Pulvinar and Amygdala, [17]): all these three areas have homologues within the avian brain (Optic tectum, Nucleus rotundus, and Amygdala, for reviews see [35], [44]–[46]). It is also worth noting that the similar functional role played by subcortical visual brain structures of different species (e.g. birds and mammals) in stimulus recognition has recently been discussed, particularly with regard to the recognition of conspecifics [14].

A further striking aspect of the present results is the convergence between face preferences displayed by the newborns of two distant vertebrate species. In the social domain, evidence of common mechanisms in distant species has already been obtained for biological motion detection, another crucial social ability, which separate studies have shown is displayed by both newborn chicks and human babies [4]-[6]. With this result in mind, some have speculated on the existence of a similar life-form perceptual detector present in different vertebrates ([47], [48] for evidence of an animate being detection device in human adults see [49]). Taken together with the results from the present study, a consistent even though still speculative picture emerges about the presence of a set of mechanisms for detecting other animals, which could involve independent mechanisms responding to biological motion and to faces. It is possible that other undiscovered biases exist and ensure preferential processing of other important aspects of conspecifics' appearance. For example, both newborn babies and face-naïve chicks react to gaze direction [27], [50], [51]. Sensitivity to eye direction may have evolved in chicks as an anti-predatory, rather than a social-affiliative, mechanism since recognizing where a predator is looking could be highly advantageous. Data in support of this hypothesis comes from the fact that chicks react to direct gaze with a longer latency to move toward visible food, which is likely to be a fear reaction in response to the predation risk [51]. This is potentially an example of a common mechanism that serves different adaptive functions in different species.

Many issues should be addressed in future studies. On the one hand, an important improvement brought about by the present work is the use, for the test of human infants and domestic chicks, of the same frequency matched stimuli employed in neuroimaging studied [15], [16]. On the other hand limitations of this study resides in the contained number of control stimuli employed. Even though adequate controls for the role of most of the potentially relevant perceptual properties are already present in the literature for both species (Tables 1 and 2), future studies may be devoted to the simultaneous control of these factors, allowing for a broader generalization of results.

Moreover, it is unclear whether the consistent behaviour observed in the two species should be considered as a product of evolutionary conservation (homology of mechanisms inherited from a common ancestor) or convergent evolution (homoplasy of mechanisms evolved independently in different species in order to cope with similar selective pressures) (see [52]). However, the issue of homology versus homoplasy is not critical for the interpretation of our results. In fact, in either case it would be parsimonious to assume that the underlying mechanisms are similar, and thus that the chick may provide a good animal model system for studying underlying mechanisms of face preferences.

Finally, it is interesting to note that previous studies have demonstrated that, in chicks, the development of the predisposition to approach naturalistic (hen-like) objects is influenced by prior exposure to certain types of non-specific experience (e.g. motor activity, manual handling, exposure to abstract visual patterns) (e.g. [53]). It is possible that also in human newborns some kind of non-specific experience could influence the degree of preference for faces, such as the release of stress hormones during the process of birth [2]. Future studies could thus investigate this issue further in human newborns by assessing the extent of non-specific experiences prior to the moment of testing.
